# The lack of association between ubiquinol‐cytochrome c reductase core protein I (*UQCRC1*) variants and Parkinson's disease in an eastern Chinese population

**DOI:** 10.1111/cns.13436

**Published:** 2020-07-14

**Authors:** Zhi‐Hao Lin, Ran Zheng, Yang Ruan, Ting Gao, Chong‐Yao Jin, Nai‐Jia Xue, Jia‐Xian Dong, Ya‐Ping Yan, Jun Tian, Jia‐Li Pu, Bao‐Rong Zhang

**Affiliations:** ^1^ Department of Neurology Second Affiliated Hospital College of Medicine Zhejiang University Hangzhou China

1

Parkinson's disease (PD) is the second most common neurodegenerative disease after Alzheimer's disease, affecting approximately 2%‐3% of the global population aged ≥65 years.^1^ The loss of dopaminergic (DA) neurons in the substantia nigral pars compacta (SNc) and intracellular α‐synuclein deposition are known as PD’s pivotal pathological characteristics. Although its etiology remains to be fully elucidated, the hypothesis that environmental and genetic factors play essential roles in the initiation and progression of PD is acknowledged worldwide.^2^ Over the past two decades, substantial progress has been made in our understanding of the genetic basis of parkinsonism through the identification of 19 monogenic disease‐causing genes. In addition, emerging evidence suggests that some monogenic genes can also increase the risk of sporadic PD through different mechanisms.^3^

Previous studies have demonstrated that mitochondrial dysfunction plays a fundamental role in the pathogenesis of PD.^4^ Moreover, mitochondrial homeostasis has been related to mutations of *Parkin*, *Pink1*, and *DJ‐1*.^5^ Ubiquinol‐cytochrome c reductase core protein I (UQCRC1) is a component of the complex III in the respiratory chain complex. Recently, Lin et al used a whole‐exome sequencing and discovered a novel candidate pathogenetic missense variant (c.941A > C p.Y314S) of the *UQCRC1* gene in a Taiwanese family with autosomal dominant parkinsonism.^6^ To further investigate the association between *UQCRC1* and PD in eastern China, we performed a *UQCRC1* genetic analysis in a cohort of sporadic PD patients and healthy controls.

We recruited 452 Chinese Han patients with sporadic PD and 450 sex‐, age‐, and ethnicity‐matched healthy controls from the Second Affiliated Hospital of Zhejiang University (Hangzhou, China) between October 2016 and January 2019. All study participants were diagnosed according to the Movement Disorder Society clinical diagnostic criteria for PD.^7^ The basic information and demographic characteristics of participants are all shown in Data S1. All participants provided written informed consent. This study was approved by the Ethics Committee of the Second Affiliated Hospital of Zhejiang University of Medicine.

Blood samples were obtained from all participants. Genomic DNA was then extracted from peripheral blood leukocytes using the QIAamp DNA Blood Mini Kit (Qiagen, Hilden, Germany) according to the manufacturer's protocol. The exon and intron‐exon boundaries of the *UQCRC1* gene were amplified by polymerase chain reaction. Detailed information on primers and annealing temperature is shown in Data S2. DNA products were directly sequenced using a Genetic Analyzer (ABI 3730XL) and analyzed with Chromas software and Mutation Surveyor software (SoftGenetics).

PD cases and healthy controls were matched for age and sex using a nonparametric test (Shapiro‐Wilk normality test, *P* < .05) and Pearson's *χ* ^2^ test, respectively. We also used Pearson's *χ* ^2^ test, *χ* ^2^ with Yates correction test or Fisher exact test to compare the allele frequencies in PD patients and healthy controls. The Hardy‐Weinberg principle was used to ascertain the normal distribution of genotype frequencies in PD patients and healthy controls. Statistical analysis was performed using SPSS20.0 software (SPSS Inc). A two‐tailed *P* < .05 was considered as threshold for statistical significance. The pathogenicity of missense variants was predicted using in silico tools including PolyPhen2, SIFT, CADD, as recommended by the American College of Medical Genetics and Genomics.^8^

In this study, we enrolled 452 sporadic PD cases and 450 healthy controls. There were no significant differences in age and sex distribution between patients and controls (Data S1). We identified three synonymous variants (p.V388=, p.A108=, and p.S473=) and four nonsynonymous variants (p.D215H, p.S4A, p.P267R, and p.N308S) of the *UQCRC1* gene. The p.A108 = and p.S4A variants were detected exclusively in PD patients (Table 1). Moreover, the homozygous p.V388 = variant and the p.P267R variant were identified separately in two PD patients. However, we did not detect the previously reported p.Y314S variant (Lin et al, 2019). Analysis using publicly available resources indicated all four nonsynonymous variants should not be deleterious (Table 2). Furthermore, the frequencies of the seven variants showed no statistically significant differences between PD patients and controls. Representative images of the variants are presented in Data S3.

**TABLE 1:** Variants identified in UQCRC1 in our study

Chromosomal position	Accession number	Variant		Number of carrying variants		PD vs controls	
		cDNA	Amino acid	PD	Controls	OR (95%CI)	*P*
Chr3:48637964	rs140583334	c.1164G>T	p.V388=	42	40	1.050 (0.667‐1.654)	0.833
Chr3:48642187	NA	c.324C>T	p.A108=	1	0	NA	1.000
Chr3:48636585	rs182453765	c.1419C>T	p.S473=	12	14	0.849 (0.388‐1.857)	0.682
Chr3:48641060	rs17080284	c.643G>C	p.D215H	29	31	0.927 (0.549‐1.565)	0.776
Chr3:48647044	NA	c.10T > G	p.S4A	1	0	NA	1.000
Chr3:48638807	rs149245457	c.800C>G	p.P267R	22	15	1.484 (0.759‐2.899)	0.245
Chr3:48638451	rs187641562	c.923A>G	p.N308S	2	3	0.662 (0.110‐3.982)	0.996

Abbreviations: CI, confidence interval; NA, not available; OR, odds ratio; *P*, *P*‐value; PD, Parkinson's disease.

**TABLE 2:** Allele frequencies and pathogenicity prediction of identified UQCRC1 missense variants

Missense variants	Freq.gnom AD(East Asian)	Freq.ExAC (East Asian)	Freq.1000G (East Asian)	SIFT score	Polyphen2 score	CADD
p.D215H	0.0291	0.0327	0.0238	0.097	0.997	25.4
p.S4A	NA	NA	NA	0.274	0.175	12.69
p.P267R	0.0410	0.0360	0.0526	0.051	0.052	9
p.N308S	0.0034	0.0042	0.002	0.258	0.093	15.41

Note

Threshold values for deleteriousness: SIFT <0.05; polyphen2 >0.86; CADD >15.

Abbreviation: NA, not available.

The *UQCRC1* gene encodes ubiquinol‐cytochrome c reductase core protein I, which is a subunit of complex III of the respiratory chain. As one of eleven subunits of complex III, UQCRC1 is a nuclear‐encoded protein localized in the inner mitochondrial membrane. Previous studies of *UQCRC1* have focused mainly on its roles in various tumors, such as malignant pleural mesothelioma and pancreatic ductal adenocarcinoma, suggesting that *UQCRC1* may contribute to carcinogenesis.^9^, ^10^ Notably, mitochondrial dysfunction also plays a crucial role in the pathogenesis of PD.^4^, ^11^ Therefore, it can be hypothesized that *UQCRC1* variants may play a role in the development of PD. Recently, *UQCRC1* was identified as a candidate pathogenic gene for PD.^6^ Nevertheless, we did not find an association between sporadic PD and *UQCRC1* in our study. Our data do not support that *UQCRC1* mutation is a common genetic factor for sporadic patients in eastern China, but our study cannot rule out its pathogenic role in PD due to the limited ethnic background and sample size. Furthermore, in consideration of the role of outer mitochondrial membrane signaling in Parkinson's disease, proteins residing in or translocating to the outer mitochondrial membrane following mitochondrial activities deserve closer attention and be important in genetic analyses for PD.^12^

In conclusion, our study indicates that *UQCRC1* may not be involved in pathogenesis of PD in eastern China. Further genetic analysis with larger sample sizes from diverse ethnic populations and functional assays are still needed to clarify the role of *UQCRC1* in the pathogenesis of PD.

### CONFLICT OF INTEREST

The authors declare no conflicts of interest.

### FUNDING INFORMATION

This work was supported by the National Natural Science Foundation of China (No. 81520108010 and 81771216) and the Primary Research and Development Plan of Zhejiang Province (No. 2020C03020).

REFERENCES1

Poewe
W
, 
Seppi
K
, 
Tanner
CM
, et al. Parkinson disease. Nat Rev Dis Primers. 2017;3:17013.2833248810.1038/nrdp.2017.132

Pang
SY
, 
Ho
PW
, 
Liu
HF
, et al. The interplay of aging, genetics and environmental factors in the pathogenesis of Parkinson's disease. Transl Neurodegener. 2019;8:23.3142831610.1186/s40035-019-0165-9PMC66966883

Reed
X
, 
Bandres‐Ciga
S
, 
Blauwendraat
C
, 
Cookson
MR
. The role of monogenic genes in idiopathic Parkinson's disease. Neurobiol Dis. 2019;124:230‐239.3044828410.1016/j.nbd.2018.11.012PMC63638644

Grunewald
A
, 
Kumar
KR
, 
Sue
CM
. New insights into the complex role of mitochondria in Parkinson's disease. Prog Neurogibol. 2019;177:73‐93.10.1016/j.pneurobio.2018.09.003302192475

Larsen
SB
, 
Hanss
Z
, 
Krüger
R
. The genetic architecture of mitochondrial dysfunction in Parkinson’s disease. Cell Tissue Res. 2018;373(1):21‐37.2937231710.1007/s00441-017-2768-8PMC60156296

Lin
CH
, 
Chen
PL
, 
Tai
CH
, et al. A clinical and genetic study of early‐onset and familial parkinsonism in taiwan: an integrated approach combining gene dosage analysis and next‐generation sequencing. Mov Disord. 2019;34(4):506‐515.3078885710.1002/mds.27633PMC65940877

Postuma
RB
, 
Berg
D
, 
Stern
M
, et al. MDS clinical diagnostic criteria for Parkinson's disease. Mov Disord. 2015;30(12):1591‐1601.2647431610.1002/mds.264248

Richards
S
, 
Aziz
N
, 
Bale
S
, et al. Standards and guidelines for the interpretation of sequence variants: a joint consensus recommendation of the American College of Medical Genetics and Genomics and the Association for Molecular Pathology. Genet Med. 2015;17(5):405‐423.2574186810.1038/gim.2015.30PMC45447539

Torricelli
F
, 
Saxena
A
, 
Nuamah
R
, et al. Genomic analysis in short‐ and long‐term patients with malignant pleura mesothelioma treated with palliative chemotherapy. Eur J Cancer. 2020;132:104‐111.3233997810.1016/j.ejca.2020.03.00210

Wang
Q
, 
Li
M
, 
Gan
Y
, et al. Mitochondrial protein UQCRC1 is oncogenic and a potential therapeutic target for pancreatic cancer. Theranostics. 2020;10(5):2141‐2157.3208973710.7150/thno.38704PMC701916011

Cowan
K
, 
Anichtchik
O
, 
Luo
S
. Mitochondrial integrity in neurodegeneration. CNS Neurosci Ther. 2019;25(7):825‐836.3074690510.1111/cns.13105PMC656606112

Lucero
M
, 
Suarez
AE
, 
Chambers
JW
. Phosphoregulation on mitochondria: Integration of cell and organelle responses. CNS Neurosci Ther. 2019;25(7):837‐858.3102554410.1111/cns.13141PMC6566066

## Supporting information

Data S1

Data S2

Data S3

## References

[1] Poewe W , Seppi K , Tanner CM , et al. Parkinson disease. Nat Rev Dis Primers. 2017;3:17013. 28332488 10.1038/nrdp.2017.13

[2] Pang SY , Ho PW , Liu HF , et al. The interplay of aging, genetics and environmental factors in the pathogenesis of Parkinson's disease. Transl Neurodegener. 2019;8:23. 31428316 10.1186/s40035-019-0165-9 PMC6696688

[3] Reed X , Bandres‐Ciga S , Blauwendraat C , Cookson MR . The role of monogenic genes in idiopathic Parkinson's disease. Neurobiol Dis. 2019;124:230‐239. 30448284 10.1016/j.nbd.2018.11.012 PMC6363864

[4] Grunewald A , Kumar KR , Sue CM . New insights into the complex role of mitochondria in Parkinson's disease. Prog Neurogibol. 2019;177:73‐93. 10.1016/j.pneurobio.2018.09.003 30219247

[5] Larsen SB , Hanss Z , Krüger R . The genetic architecture of mitochondrial dysfunction in Parkinson’s disease. Cell Tissue Res. 2018;373(1):21‐37. 29372317 10.1007/s00441-017-2768-8 PMC6015629

[6] Lin CH , Chen PL , Tai CH , et al. A clinical and genetic study of early‐onset and familial parkinsonism in taiwan: an integrated approach combining gene dosage analysis and next‐generation sequencing. Mov Disord. 2019;34(4):506‐515. 30788857 10.1002/mds.27633 PMC6594087

[7] Postuma RB , Berg D , Stern M , et al. MDS clinical diagnostic criteria for Parkinson's disease. Mov Disord. 2015;30(12):1591‐1601. 26474316 10.1002/mds.26424

[8] Richards S , Aziz N , Bale S , et al. Standards and guidelines for the interpretation of sequence variants: a joint consensus recommendation of the American College of Medical Genetics and Genomics and the Association for Molecular Pathology. Genet Med. 2015;17(5):405‐423. 25741868 10.1038/gim.2015.30 PMC4544753

[9] Torricelli F , Saxena A , Nuamah R , et al. Genomic analysis in short‐ and long‐term patients with malignant pleura mesothelioma treated with palliative chemotherapy. Eur J Cancer. 2020;132:104‐111. 32339978 10.1016/j.ejca.2020.03.002

[10] Wang Q , Li M , Gan Y , et al. Mitochondrial protein UQCRC1 is oncogenic and a potential therapeutic target for pancreatic cancer. Theranostics. 2020;10(5):2141‐2157. 32089737 10.7150/thno.38704 PMC7019160

[11] Cowan K , Anichtchik O , Luo S . Mitochondrial integrity in neurodegeneration. CNS Neurosci Ther. 2019;25(7):825‐836. 30746905 10.1111/cns.13105 PMC6566061

[12] Lucero M , Suarez AE , Chambers JW . Phosphoregulation on mitochondria: Integration of cell and organelle responses. CNS Neurosci Ther. 2019;25(7):837‐858. 31025544 10.1111/cns.13141 PMC6566066

