# Supplementary material for: The lack of association between ubiquinol‐cytochrome c reductase core protein I (UQCRC1) variants and Parkinson's disease in an eastern Chinese population
Source: CNS Neurosci Ther. 2020 Jul 14;26(9):990–2. doi: 10.1111/cns.13436 (PMC7415203; doi:10.1111/cns.13436)
Supplement: Supplementary file 1 — Data S1 [file CNS-26-990-s001.docx]

Data 1 Demographic characteristics of study participants

| variables | PD group  （n=452） | Control group  （n=450） | *p* |
| --- | --- | --- | --- |
| Sex(male/female)  Age(y), mean±SD | 241/211  57.43±11.59 | 232/218  56.54±9.27 | 0.596  0.174 |

Key: PD, Parkinson’s disease；SD, standard deviation; *p*, *p*-value
