# Supplementary material for: The lack of association between ubiquinol‐cytochrome c reductase core protein I (UQCRC1) variants and Parkinson's disease in an eastern Chinese population
Source: CNS Neurosci Ther. 2020 Jul 14;26(9):990–2. doi: 10.1111/cns.13436 (PMC7415203; doi:10.1111/cns.13436)

Supplementary table 2 Primers for sequence analysis

| Primers | Forward sequence | Reverse sequence | Annealing temperature |
| --- | --- | --- | --- |
| Exon 1 | TCAGAGCCAGTCAGCCAAAGCC | GCGAGATACCTTTCCCCGCCCTC | 66.8℃ |
| Exon 2 | CGATCCCCTGACCGAGTCC | AACCCAAACCACAAACGGGAA | 60.6℃ |
| Exon 3 | TGCCCCTGTTTTCCAAGCAT | CATCAGATACGTGCCCACTCAG | 60.6℃ |
| Exon 4 | TGCCATGGTTTTGGTCACCT | AGCTGCAAAGCCATACGCTA | 58.2℃ |
| Exon 5 | ACCTGGCACCTAAACATGTCC | CAGGAAAATAACCCCGACAGC | 61.6℃ |
| Exon 6 | GCATGTACCCCAACTCACTCC | AGGACCTCGGCTTTGATAACCA | 63.8℃ |
| Exon 7-8 | CCTCTGCCTGTCTGTGAATCG | CCCTCTTGTCTTCAACGCACAC | 63.8℃ |
| Exon 9-10 | CCATCATCGGCCACTATGACT | GGCAAGATGTACTCTACCCCTC | 60.6℃ |
| Exon 11 | CAGGTCCACAGCCCATCACT | TGCCCTCCCACTATGCTACCC | 64.5℃ |
| Exon 12 | CATCCCTGGTGTCCTAGGCA | TCATGTTCTGGCCCTTGAAGC | 61.8℃ |
| Exon 13 | AGGTGCCTTCATCAATACCCT | CTGGCTGAGGAACTCCCCTA | 62.9℃ |


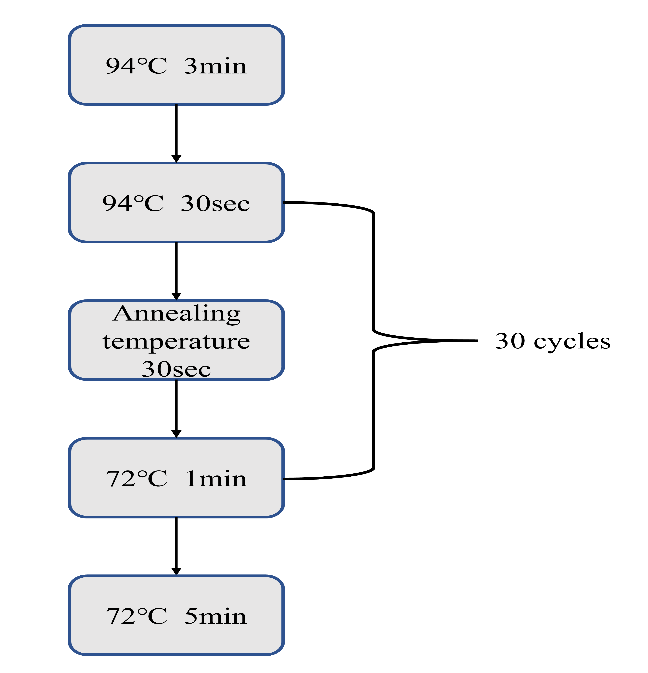

Supplement: Supplementary file 2 — Data S2 [file CNS-26-990-s002.docx]
