# Supplementary figures and images for: The lack of association between ubiquinol‐cytochrome c reductase core protein I (UQCRC1) variants and Parkinson's disease in an eastern Chinese population
Source: CNS Neurosci Ther. 2020 Jul 14;26(9):990–2. doi: 10.1111/cns.13436 (PMC7415203; doi:10.1111/cns.13436)

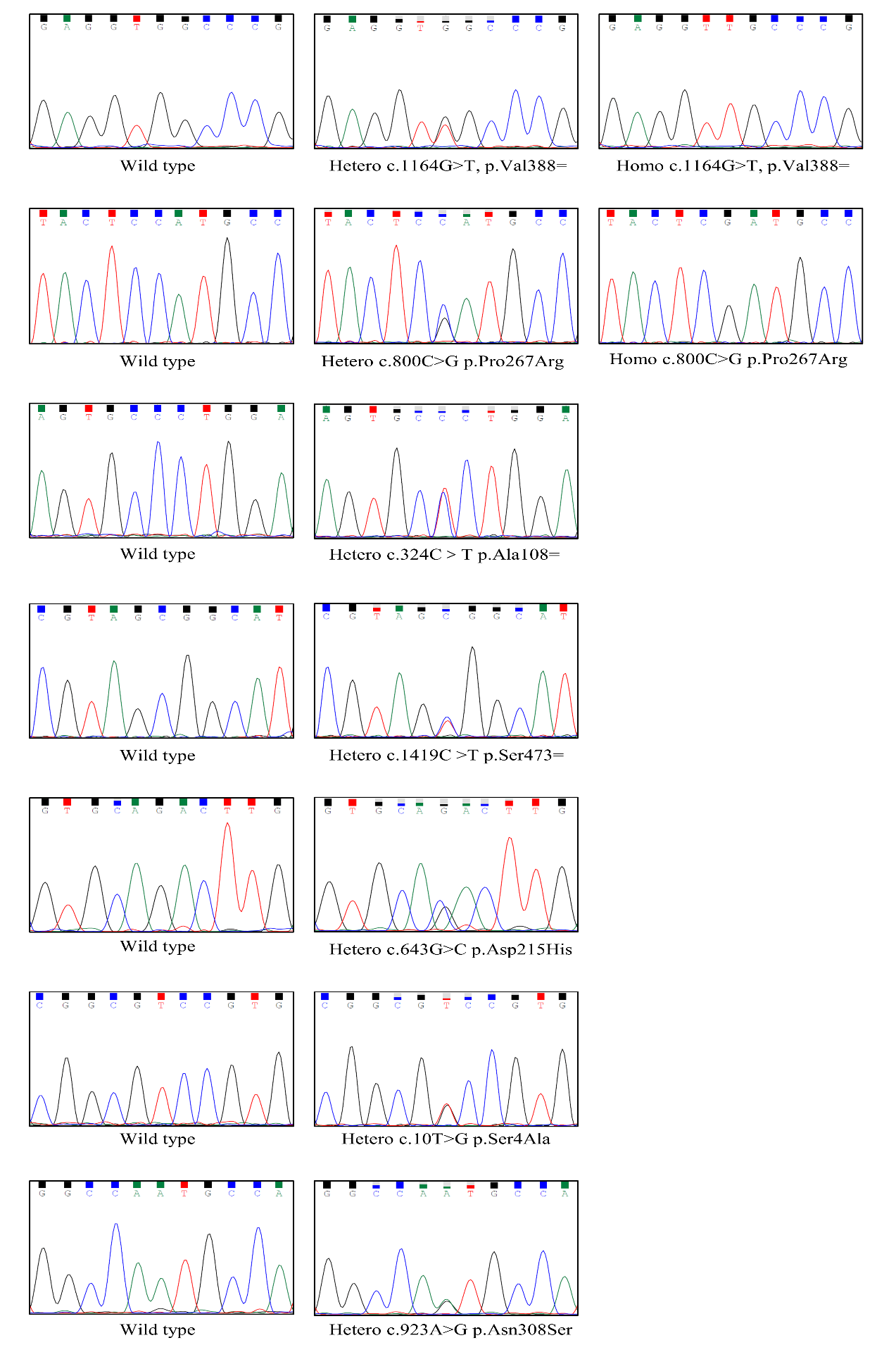
Supplementary data 3: representative image of variants

Supplement: Supplementary file 3 — Data S3 [file CNS-26-990-s003.docx]
